# Nitrogen-Doped Carbonaceous Materials for Removal of Phenol from Aqueous Solutions

**DOI:** 10.1100/2012/297654

**Published:** 2012-04-19

**Authors:** Magdalena Hofman, Robert Pietrzak

**Affiliations:** Laboratory of Applied Chemistry, Faculty of Chemistry, A. Mickiewicz University, Grunwaldzka 6, 60-780 Poznań, Poland

## Abstract

Carbonaceous material (brown coal) modified by pyrolysis, activation, and enrichment in nitrogen, with two different factor reagents, have been used as adsorbent of phenol from liquid phase. Changes in the phenol content in the test solutions were monitored after subsequent intervals of adsorption with selected adsorbents prepared from organic materials. Significant effect of nitrogen present in the adsorbent material on its adsorption capacity was noted. Sorption capacity of these selected materials was found to depend on the time of use, their surface area, and pore distribution. A conformation to the most well-known adsorption isotherm models, Langmuir, and Freundlich ones, confirms the formation of mono- and heterolayer solute (phenol) coverage on the surface of the adsorbent applied herein. The materials proposed as adsorbents of the aqueous solution contaminants were proved effective, which means that the waste materials considered are promising activated carbon precursors for liquid phase adsorbents for the environmental protection.

## 1. Introduction

Phenol and its derivatives are frequent and toxic byproducts in industrial processes and their disposal is of great concern from the viewpoint of the environment protection [1]. The presence of phenol and its derivatives in natural water sources is a serious threat to human health and general water quality [2, 3]. Phenolic compounds are harmful to living organisms even at low concentrations due to their toxicity and carcinogenic properties. Phenol is a combustible compound highly soluble in water, oils, carbon disulfide and numerous organic solvents [4, 5]. It is characterized, by a typical pungent sweet, medicinal as well as tar-like odour [6]. Phenol has been registered as priority pollutant by the US Environmental Protection Agency (USEPA) with a permissible limit of 0.1 mg/L in wastewater [7].

Several ways have been developed to remove phenol from wastewater, including electrochemical oxidation, chemical coagulation, solvent extraction, membrane separation, and photocatalytic degradation [8–13]. It is believed that physisorption, due to dispersive forces, is the mechanism behind the use of activated carbons for removal of phenol from aqueous solutions. Furthermore, a large number of other contaminants may be removed from a liquid or gas stream during their passage through an activated carbon bed.

The adsorption properties of activated carbon are attributed to its physical and chemical structure. Previous studies have shown that the adsorption character of activated carbon depends on the surface characteristics [12–18]. Materials of organic origin modified in different processes such as pyrolysis, activation, or enrichment in a specific heteroatoms (nitrogen, sulphur, phosphorus) show a highly developed porous structure, which allows their application as adsorbents, both from the gas and liquid phases [16–20].

Surface chemical composition is mainly determined by the oxygen functional groups representing either Brönsted acidic or basic sites. The effect of surface functional groups modification on the adsorption behaviour of activated carbons has been of great interest over the last years. Increasingly stricter air and water quality legislation have prompted the interest in preparation of activated carbons with certain surface functional groups for removal of specific contaminants [21]. The nature and concentration of these surface groups may be modified by various pre- or postactivation treatment methods [22, 23]. Furthermore, the surface groups play a vital role by inducing the formation of chemical bonding between adsorbate and adsorbent, especially in the process of adsorption of organic compounds. Thus, introduction of other heteroatoms is of great interest as well. Recently, much attention has been paid to nitrogen functionalities in carbonaceous materials because of their interesting textural properties and high density of surface nitrogen sites of the Lewis basic character. Several modification processes have been reported and among them those based on as ammonia, urea, and other nitrogen- containing substances [24]. It has been established earlier that the efficient method of enrichment in nitrogen is ammoxidation, involving a simultaneous oxidation of the precursor [25–34], as well as nitrogenation by NO [35–37].

X-ray photoelectron spectroscopy (XPS) and FT-IR/PAS studies of the organic origin samples enriched in nitrogen proved that the chemical character of surface nitrogen groups formed upon ammoxidation differs from those formed as a result of nitrogenation with nitrogen(II) oxide. The nitrogen groups dominant on the surface of the materials subjected to ammoxidation are pyrrole, pyridine, and amine ones [26, 27], but when nitrogen(II) oxide was the source of nitrogen, the dominant nitrogen species are amide groups and nitrates [35]. As follows from the results of our earlier studies, the samples enriched in nitrogen by ammoxidation or nitrogenation differ in the catalytic properties [29–33] and capacities of adsorption from gas phase [33, 34], according to the chemical character of the nitrogen species identified on their surface.

In this respect, the aim of this paper was to explore the effectiveness of above mentioned materials as a low-cost adsorbent of liquid phase contaminants. Brown coal was modified by its pyrolysis, activation, and enrichment in nitrogen by two different methods generating surface groups with various chemical character and examined for the removal of phenol from aqueous solution. The equilibrium and kinetic data of adsorption were then studied to understand the adsorption process.

## 2. Experimental

### 2.1. Materials and Preparations of Adsorbents

The starting material applied was brown coal (B). It was ground in a roller mill and sieved to a uniform size of 1.5–2.5 mm. The samples obtained were subjected to pyrolysis, modification by nitrogen reagent, and to physical activation.

Pyrolysis (K) was carried out in a horizontal furnace under a stream of argon at the flow rate of 170 mL/min. The sample was heated (5°C/min) from room temperature to the final temperature of pyrolysis 700°C at which it was kept for 60 min and then it was cooled in inert atmosphere.

The activation process (A) was performed at 800°C in a laboratory furnace, to about 50% of burn-off. Water was fed by two micro-feeding pumps, the steam leaving the reactor was directed to the cooler in which it was liquefied and the gases formed in the reaction after passing the cooler and gas meter were combusted in a gas burner. The samples were heated (10°C/min) from room temperature to the final activation temperature, kept at this temperature for 90 min, and cooled to room temperature.

Modification by nitrogen reagent was performed by two methods. Ammoxidation (N) was carried out using a mixture of ammonia and air at a volume ratio of 1 : 3 (250/750 mL/min) in a flow reactor at 270°C for 5 h [25–27]. In the process of nitrogenation, the samples were exposed to nitrogen(II) oxide (620 mL/min) in a flow reactor at 300°C for 2 h [35].

### 2.2. Batch Equilibrium and Kinetic Studies

In adsorption equilibrium, experiments were conducted in a set of 250 mL Erlenmeyer flasks. Phenol solutions with different initial concentrations at 100–500 mg/L were added into the flasks and the total volume of the solution was 200 mL in each flask. Equal masses of adsorbents (0.5 g) were added to phenol solutions and each sample was kept on magnetic stirrer of 120 rpm at 25 ± 1°C for different time intervals. A similar procedure was followed for another set of Erlenmeyer flasks containing solutions of the same phenol concentrations without activated carbon to be used as blank samples. The flasks were then removed from stirring, centrifuged, and obtained solution was analysed using a double beam UV-Vis spectrophotometer (Varian Cary 100 Bio) at 510 nm wavelength. The samples were filtered prior to analysis in order to minimize interference of carbon grains in analysis. Each experiment was repeated twice under identical conditions.

The amount of adsorption at equilibrium, *Q*_*e*_ (mg/g), was calculated according to the following equation:

(1) $$Q_{e}=\frac{V\left(C_{o}-C_{e}\right)}{W},$$

where *C*_0_ and  *Ce* (mg/L) are the liquid-phase concentrations of phenol at the initial and equilibrium state, respectively; *V*(L) is the volume of the solution, and *W*(g) is the mass of dry adsorbent used.

### 2.3. Instruments and Characterization of the Adsorbents

#### 2.3.1. Elemental Analysis

The chemical compositions of the samples investigated were established on an elemental analyser CHNS Perkin Elmer 2400 series II.

#### 2.3.2. Surface Oxygen Groups

The content of surface oxide functional groups was determined by the Boehm method [38].

#### 2.3.3. Porous Structure

The pore structure of activated carbons was characterised on the basis of low-temperature nitrogen adsorption-desorption isotherms measured on a sorptometer ASAP 2010, manufactured by Micromeritics Instrument Corp. (USA). Before the isotherm measurements, the samples were outgassed at 300°C for 10 h. Surface area and pore size distribution were calculated by BET and BJH methods, respectively. Total pore volume and average pore diameter were determined as well. Micropore volume and micropore surface area were calculated using the *t*-plot method.

## 3. Results and Discussion

### 3.1. Physicochemical Characteristic of Adsorbents

Investigation of the elemental composition of the adsorbents studied proved that modification of the active carbon precursor by ammoxidation (BKN) or nitrogenation (BK(NO)) leads to considerable changes in the content of nitrogen relative to that of the unmodified sample (BK) [26, 35]. The content of nitrogen in the samples modified by nitrogenation (BK(NO)-3.9 wt.%) is a bit lower [35] than that in the sample modified by ammoxidation (BKN-9.8 wt.%) [26]. Moreover, textural parameters of the adsorbents presented in our previous work [26, 35] indicate that nitrogen modified samples (BKN-212 m^2^/g and BK(NO)-112 m^2^/g) are characterized by lower values of surface area and total pore volume as compared with BK-224 m^2^/g. The above is a consequence of interaction of the modifying agent, that is, NH_3_/air mixture and nitrogen(II) oxide with the modified material. Activation of the above-mentioned samples (BKNA and BK(NO)A) has a beneficial effect on their structural parameters, endowing them with well-developed surface area (800 and 524 m^2^/g, resp.) with a dominant contribution of micropores (Vmic/Vt = 0.75 and 0.71, resp.).

Acid-base character of discussed materials was determined and summarized in Table 1. Additionally, the nitrogen content (wt. %) is presented in the same table for a better understanding of the nitrogen influence on the above properties. In the pyrolysed sample (BK) the majority of surface oxide groups reveal acidic character. Modification of the above sample leads to a significant increase in the total content of surface oxides. Furthermore, for nitrogenised sample (BK(NO)) the majority of surface oxides exhibit acidic character, whereas analogous-activated samples for example, BK(NO)A, exhibit higher content of basic groups. A significant increase in the content of basic groups as compared with pyrolysed material probably results from the presence of numerous oxygen-nitrogen complexes generated on the surface of the samples in the process of their modification.

It is worth noting that the nitrogenised sample (BK(NO)) has the lowest pH value from among all samples investigated herein.

### 3.2. Effect of Initial Concentration

**Figure 1:**
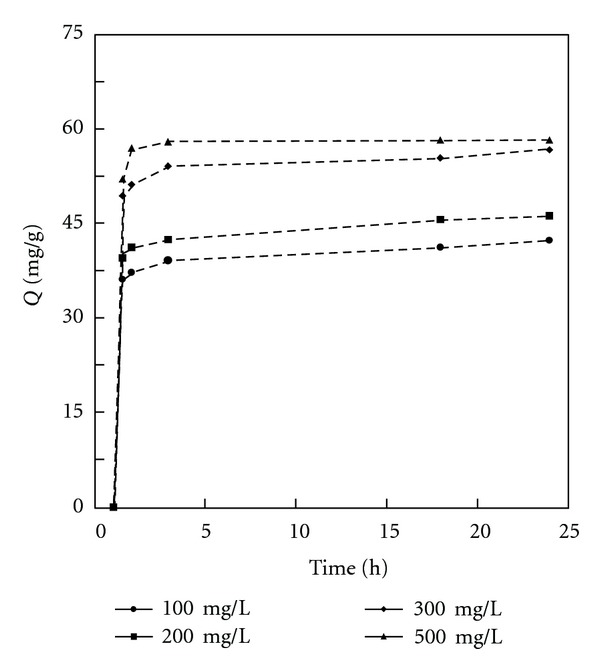
Dynamics of phenol uptake by BK for various initial phenol concentrations.

Figure 1 describes the effect of phenol initial concentrations on the percentage of its removal by BK. This sample was chosen for the process of optimisation of the adsorption conditions because it would be considered as the standard adsorbent containing small amount of nitrogen/oxygen groups and having the most ordered surface. It is evident that an increase in the initial phenol concentration results in increased phenol uptake. The phenol removal curves exhibit single, smooth, and continuous shapes. The shape indicates the formation of phenol molecule monolayer covering the outer surface of the adsorbent. The isotherms shape shows a tendency to level out at high adsorbate concentrations. The maximum adsorption capacity at equilibrium (*Q*) increased from 42.3 to 58.3 mg/g with the initial phenol concentrations increasing from 100 to 500 mg/L. According to there literature, there are two consecutive mass transportation steps associated with the adsorption of solute from solution by a porous adsorbent [39]. At first the adsorbate molecules migrate into the adsorbent pores from the solution and then it is adsorbed at the active sites inside the adsorbent particle. This process requires relatively long contact time.

### 3.3. Effect of Adsorbent Surface Properties

It is known that phenol adsorption onto activated carbon may occur via a complex interplay of electrostatic and dispersion interactions with three possible mechanisms [5, 40].

The first one is *π*-*π* dispersion interaction between the phenol aromatic ring and the delocalised *π* electrons present in the aromatic structure of the graphite layers. The second is via hydrogen bond formation, whereas the last one is electron donor-acceptor complex formation at the carbon surface, where the oxygen of the surface carbonyl group acts as the electron donor and the phenol aromatic ring as the acceptor [41, 42]. What is more, electrostatic interactions can play a significant role if phenol is predominately in the phenolate ion form that can interact with the charged adsorbent surface [40]. 

Furthermore, phenol is considered as a weak acid, thus phenol adsorption should be enhanced in activated carbons with basic surface functional groups [21]. It has been shown that adsorption of phenol is dependent not only on porosity but also on the presence of surface groups [21, 43]. Introduction of acidic functional groups may cause removal of *π*-electrons from the carbon matrix, leading to a decrease in the strength of the interactions between the aromatic ring of the phenol molecule and the carbon basal planes [44].

**Figure 2:**
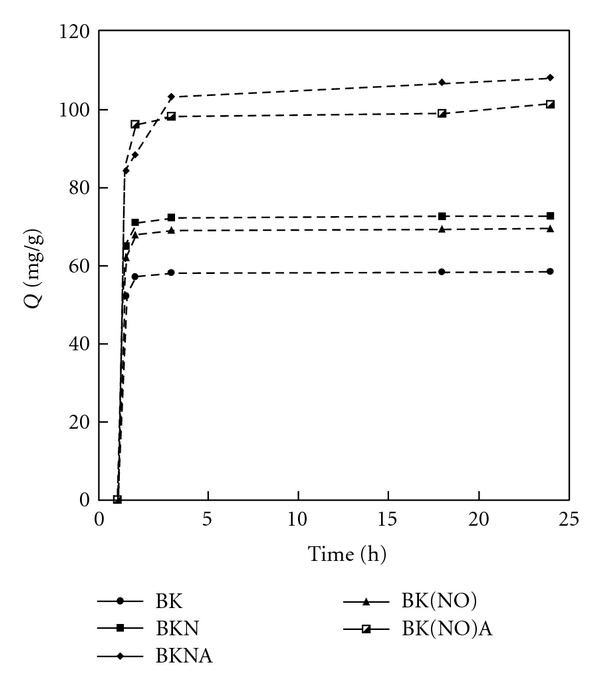
Fractional phenol removals as a function of time (*C*  initial = 500 mg/L; 25°C).

In this respect the adsorption of phenol onto the adsorbents prepared form brown coal precursor enriched with nitrogen by two different reagents as well as by its steam activation at the most representative initial phenol concentration of 500 mg/L was studied as a function of contact time in order to determine the equilibrium time (Figure 2).

It can be noticed that the saturation curves rise sharply in the initial stages, indicating that there are plenty of readily accessible adsorption sites. All curves reach a plateau indicating that the adsorbent is saturated at this level. As indicated in Figure 2, the contact time needed to reach equilibrium for phenol solutions with initial concentrations of 500 mg/L was 3 h in all investigated processes. 

The lowest sorption capacity was observed for sample BK. As follows from the saturation curves, the adsorption capacity of ammoxidised samples (N) is greater than that of those modified by nitrogenation (NO). Most probably it follows from the presence of nitrogen groups being of basic character for the ammoxidised samples [26]. In the same time acidic groups for the samples modified by nitrogenation [35]. It should be noted that for both, ammoxidised as well as nitrogenated materials, the higher adsorption capacity is obtained for activated carbon (BKNA and BK(NO)A), which holds true for every time range applied in this study.

The enhancement of phenol adsorption observed for the nitrogen modified samples might be attributed to the nitrogen functional groups presence in the samples. The *π*-*π* dispersion forces were increased in adsorbents enriched with nitrogen, due to the corresponding increase in electronic density of basal surface resulting in the enhancement of the adsorption process [41]. It can be assumed that in the present study the nitrogen functionalities improved the phenol adsorption capacity of activated carbons.

### 3.4. Adsorption Isotherms

Adsorption isotherm is important to describe the solute interaction with adsorbents at a constant temperature and its concentration in the equilibrium solution. It provides essential physiochemical data for assessment of the applicability of the adsorption process as a complete unit operation [45, 46]. Langmuir and Freundlich isotherm models are widely used to investigate the adsorption process [45, 47–49]. The Langmuir isotherm was developed assuming that the adsorption process will only take place at specific homogenous sites within the adsorbent surface with a uniform distribution of energy level. Once the adsorbate is attached to the site, no further adsorption can take place there, which implies that the adsorption process is monolayer in nature. The Freundlich isotherm, contrarily to that of Langmuir type, was based on the assumption that the adsorption occurs on heterogeneous sites with a nonuniform distribution of energy level. It describes reversible adsorption and is not restricted to the formation of monolayer [45].

The linear forms of Langmuir and Freundlich equations are represented by (2) and (3), respectively:

(2) $$\frac{C_{e}}{Q_{e}}=\frac{1}{K_{L}}+\left(\frac{a_{L}}{K_{L}}\right)C_{e},$$

(3) $$\log Q_{e\;}=\log K_{F}+\left(\frac{1}{n}\right)\log C_{e},$$

where *Q*_*e*_ is the amount of the adsorbate adsorbed at equilibrium (mg/g), *C*_*e*_ is equilibrium concentration of the adsorbate (mgl/L). *K*_*L*_ (L/g) and *a*_*L*_ (L/mg) are Langmuir isotherm constants.

For the Langmuir isotherm, plots of *C*_*e*_/*Q*_*e*_ versus *C*_*e*_ are linear and have a slope *a*_*L*_/*K*_*L*_ and the intercept 1/*K*_*L*_, where *K*_*L*_/*a*_*L*_ gives the Langmuir constant related to a maximum adsorption capacity at the monolayer, *Q*_0_ (mg/g). As for the Freundlich isotherm, a plot of log*Q*_*e*_ versus log*C*_*e*_ enables the determination of constant *K*_*F*_ and exponent 1/*n*. *K*_*F*_ is the Freundlich constant (mg/g)(L/mg)^1/*n*^ and 1/*n* is dimensionless heterogeneity factor.

**Figure 3:**
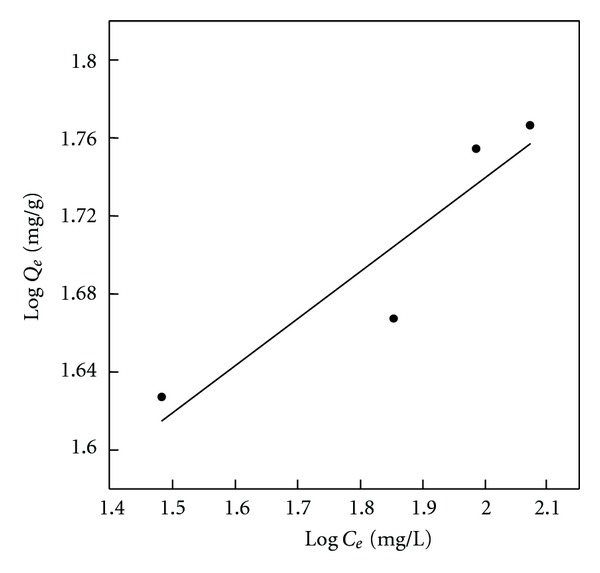
Freundlich isotherm plots of phenol adsorption onto BK (initial concentrations = 100–500 mg L^−1^, 25°C).

**Figure 4:**
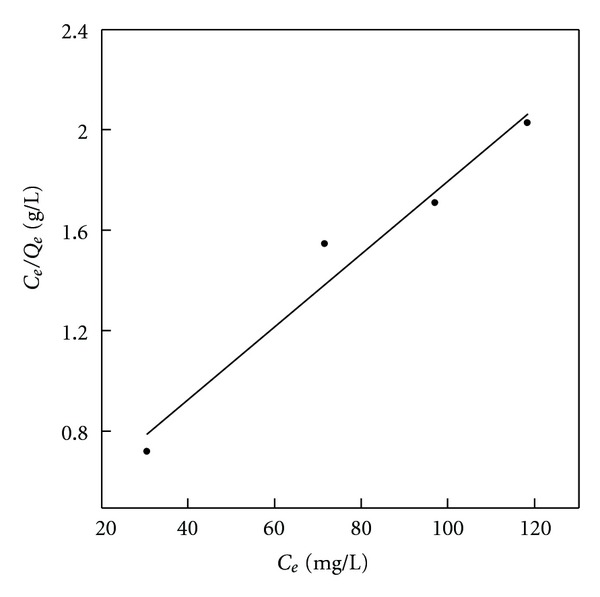
Langmuir isotherm plots of phenol adsorption onto BK (initial concentrations = 100–500 mg L^−1^, 25°C).

The linear plots of Langmuir isotherm and Freundlich isotherm for phenol adsorption onto BK as the standard adsorbent containing a small amount of nitrogen/oxygen and the most ordered surface are presented in Figures 3 and 4, respectively. It is worth noticing that the highest n factor as well as *K*_*F*_ value, which are taken as indicators of adsorption capacity, are observed for the sample BKN (Table 2). The positive impact of nitrogen presence in adsorbent surface on the *K*_*F*_ value is demonstrated especially for BKN and BKNA samples.

Both, Langmuir and Freundlich equations are reasonably applicable to all investigated adsorbents with correlation coefficients (*R* ^2^) in the range of 0.919–0.990 (Table 2). This observation means that the phenol adsorption in this study is possible by formation of mono and heterolayers on the adsorbent surface. It can be explained by the presence of surface functional groups in different amounts, which may induce differences in the energy levels of the active sites available on the adsorbent surface, thus affecting adsorption capacity. Similar findings have been reported earlier [45, 49].

### 3.5. Kinetic Studies

The kinetic model proposed by Lagergren and the model of intramolecular diffusion are applied to determine the factors influencing the reaction rate and the mechanism of phenol adsorption [45]. Linear forms of pseudo-first- and pseudo-second-order kinetic equations are given in (4) and (5), respectively:

(4) $$\ln\left(Q_{e}-Q_{t}\right)=\ln Q_{e}-k_{1}t,$$

(5) $$\frac{t}{Q_{t}}=\frac{1}{k_{2}Q_{e}^{2}}+\;\left(\frac{1}{Q_{e}}\right)t,$$

where *Q*_*t*_ is the amount of solute adsorbed per unit weight of adsorbent (mg/g), *k*_1_ is the rate constant of pseudo-first-order sorption (1/h), and *k*_2_ is the rate constant of pseudo-second-order sorption (g/h mg).

**Figure 5:**
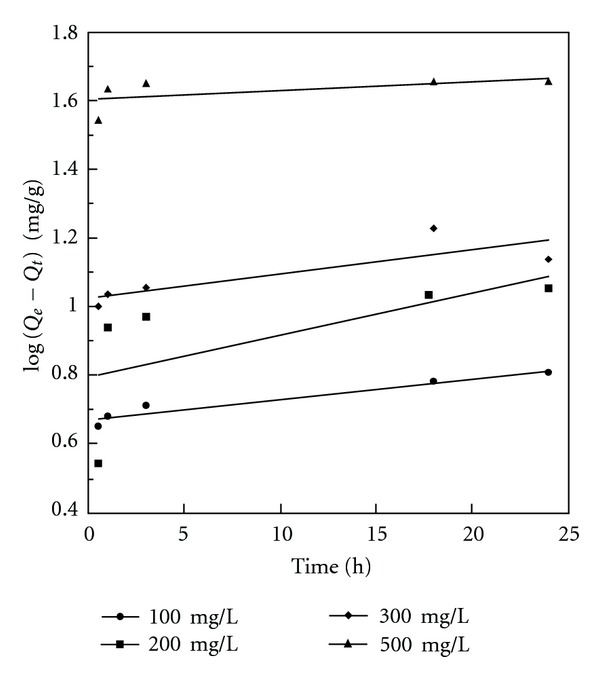
Pseudo-first-order kinetics for adsorption of phenol onto BK at 25°C.

**Figure 6:**
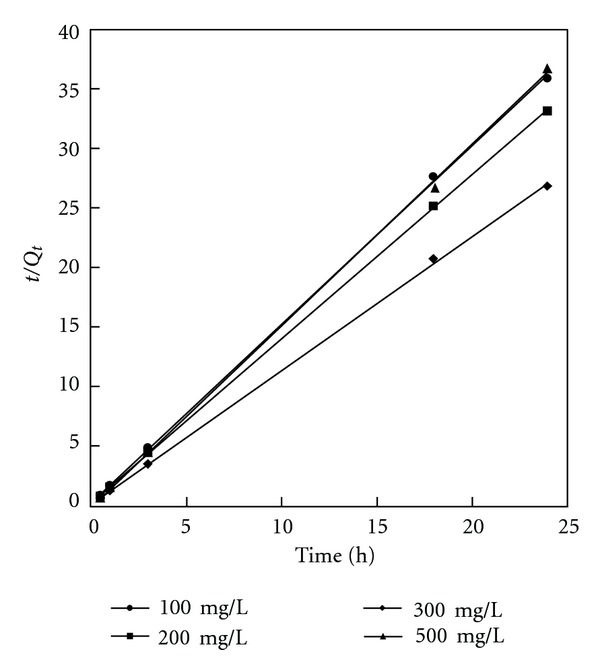
Pseudo second-order kinetics for adsorption of phenol onto BK at 25°C.

Linear plots of pseudo-first- and pseudo-second-order kinetic models of phenol adsorption onto BK are given in Figures 5 and 6, respectively, whereas the appropriate constants at various initial phenol concentrations are given in Table 3.

For the pseudo-first-order model, the rate constant slightly increases with increasing initial phenol concentration. However, the correlation coefficients obtained for this kinetic model are in the range of 0.863–0.983. Significantly, the *R* ^2^ values for the pseudo-second-order model are close to unity indicating that this model is more suitable for the adsorption process studied.

The applicability of both kinetic models was further verified by normalized standard deviation Δ*Q* (%), defined as (6):

(6) $$\Delta Q\left(\%\right)=\;100\times \sqrt{\overset{}{\underset{}{\sum }}\;\frac{{\left[\left(Q_{\exp}-Q_{\text{cal}}\right)/Q_{\exp}\right]}^{2}}{\left(n-1\right)}},$$

where the subscripts “exp” and “cal” refer to the experimental and calculated values, respectively, and *n* is the number of data points. The higher the value of *R* ^2^ and the lower the value of Δ*Q* (%), the better the fit is. The values of  Δ*Q* (%) for pseudo-first-order model are significantly high. Thus, it can be assumed that the pseudo-second-order adsorption mechanism was predominant in the adsorption studied, and that the overall rate of phenol adsorption process appeared to be controlled by the chemisorption process. Similar regularity has been also observed in the adsorption of phenol on activated carbons prepared from organic waste materials of other types [45, 47–50].

## 4. Conclusions

The adsorbents prepared from brown coal can be used for the effect removal of phenol from aqueous solutions. According to the results obtained, BKNA samples show the highest adsorption capacity of phenol. Moreover, adsorption capacities of the nitrogen modified adsorbents (BKN and BK(NO)) increase relative to that of the unmodified material (BK) as a result of hydrogen-bonding interaction with nitrogen groups and the adsorbate molecules. A conformation to the most well-known adsorption isotherm models, Langmuir and Freundlich ones, confirms the formation of mono- and heterolayer solute (phenol) coverage on the surface of the adsorbents applied herein. The results indicated that the pseudo-second-order equation provided the better correlation for the adsorption kinetics data. The adsorbing performance of N-doped activated carbons prepared from brown coal (BKNA and BK(NO)A) was remarkable as compared to those of other materials, indicating these materials as promising for phenol compounds removal. These findings are important to enhance the credibility of carbonaceous materials as one of the most promising activated carbon precursors for liquid phase adsorbents.

## Figures and Tables

**Table 1:** Acid-base properties of investigated adsorbents (m mol/g) and nitrogen content (wt.%).

Sample	*N*	Acidic groups	Basic groups	Total content of surface oxides	pH
BK	0.0	0.84	0.12	0.96	6.1
BKN	9.8	1.04	1.12	2.16	6.3
BKNA	1.6	1.39	1.23	2.62	7.2
BK(NO)	3.9	1.52	1.13	2.65	5.9
BK(NO)A	1.4	1.26	1.55	2.81	8.4

**Table 2:** Freundlich and Langmuir parameters for the adsorption of phenol.

Adsorbent	Freundlich isotherm parameters			Langmuir isotherm parameters		
	*K*_*F*_(mg/g)(L/mg)^1/*n*^	*n*	*R* ^2^	*K*_*L*_ (L/g)	*a*_*L*_· (L/mg)	*R* ^2^
BK	28.08	4.151	0.950	2.848	0.041	0.963
BKN	40.07	5.767	0.935	4.389	0.055	0.990
BKNA	30.78	3.582	0.927	1.740	0.014	0.919
BK(NO)	22.94	2.689	0.936	2.212	0.205	0.963
BK(NO)A	13.73	1.454	0.987	1.305	0.047	0.920

*K*_*F*_, *n*—Freundlich isotherm constants; *K*_*L*_, *a*_*L*_—Langmuir isotherm constants, *R*—correlation coefficient.

**Table 3:** Pseudo-first-order and pseudo-second-order constants for the adsorption of phenol on BK at 25°C.

*C*_*o*_ (mg/L)	Pseudo-first-order kinetic model				Pseudo-second-order kinetic model			
	*Q*_*e*_ cal (mg/g)	*k*_1_ (1/h)	*R* ^2^	Δ*Q* (%)	*Q*_*e*_ cal (mg/g)	*k*_2_ (1/h)	*R* ^2^	Δ*Q* (%)
100	4.69	0.006	0.863	47.20	40.00	0.65	0.992	16.17
200	6.25	0.012	0.824	49.30	43.47	0.71	0.998	18.58
300	10.29	0.013	0.983	56.21	53.48	0.98	0.988	21.01
500	40.42	0.003	0.932	37.45	39.37	1.12	0.991	19.55

*C*_*o*_: starting concentration; *Q*_*e*_ cal: amount of adsorbate adsorbed at equilibrium; Δ*Q*: applicability; *R*: correlation coefficient; *k*_1_: rate constant of pseudo-first-order sorption; *k*_2_: rate constant of pseudo-second-order sorption.
